# GFP Loss-of-Function Mutations in *Arabidopsis thaliana*

**DOI:** 10.1534/g3.115.019604

**Published:** 2015-07-06

**Authors:** Jason L. Fu, Tatsuo Kanno, Shih-Chieh Liang, Antonius J. M. Matzke, Marjori Matzke

**Affiliations:** Institute of Plant and Microbial Biology, Academia Sinica 128, Section 2, Academia Road, Nangang District, Taipei 115, Taiwan

**Keywords:** *Arabidopsis thaliana*, green fluorescent protein, protein stability, protein structure, reporter gene

## Abstract

Green fluorescent protein (GFP) and related fluorescent proteins are widely used in biological research to monitor gene expression and protein localization in living cells. The GFP chromophore is generated spontaneously in the presence of oxygen by a multi-step reaction involving cyclization of the internal tripeptide Ser65 (or Thr65)-Tyr66-Gly67, which is embedded in the center of an 11-stranded β-barrel structure. Random and site-specific mutagenesis has been used to optimize GFP fluorescence and create derivatives with novel properties. However, loss-of-function mutations that would aid in understanding GFP protein folding and chromophore formation have not been fully cataloged. Here we report a collection of ethyl methansulfonate–induced *GFP* loss-of-function mutations in the model plant *Arabidopsis thaliana*. Mutations that alter residues important for chromophore maturation, such as Arg96 and Ser205, greatly reduce or extinguish fluorescence without dramatically altering GFP protein accumulation. By contrast, other loss-of-fluorescence mutations substantially diminish the amount of GFP protein, suggesting that they compromise protein stability. Many mutations in this category generate substitutions of highly conserved glycine residues, including the following: Gly67 in the chromogenic tripeptide; Gly31, Gly33, and Gly35 in the second β-strand; and Gly20, Gly91, and Gly127 in the lids of the β-barrel scaffold. Our genetic analysis supports conclusions from structural and biochemical studies and demonstrates a critical role for multiple, highly conserved glycine residues in GFP protein stability.

Green fluorescent protein (GFP) is a genetically encoded reporter that is intrinsically fluorescent and detectable in the absence of a substrate or cofactor (Tsien 1998; Zacharias and Tsien 2006). The chromophore of wild-type GFP, which is 238 amino acids in length, forms through autocatalytic cyclization and oxidation of the internal tripeptide Ser65-Tyr66-Gly67 of the GFP primary sequence (Zacharias and Tsien 2006; Stepanenko *et al.* 2013). In a commonly used variant named Thr65-GFP, Ser65 has been replaced by threonine, resulting in a six-fold increase in fluorescence through suppression of one of two absorption peaks exhibited by wild-type GFP (Heim *et al.* 1995; Ormö *et al.* 1996; Brejc *et al.* 1997). Determination of the crystal structures of wild-type GFP and Thr65-GFP revealed a β-barrel structure comprising 11 β-strands with lids flanking both sides and an internally positioned α-helix that harbors the chromophore (Yang *et al.* 1996; Ormö *et al.* 1996).

Various mutational strategies have been used to modify and optimize GFP. Improvements achieved in this way include increasing the fluorescence intensity by facilitating protein folding, creating different color variants, reducing the pH sensitivity, increasing thermostability, and minimizing the propensity to form dimers (Zacharias and Tsien 2006; Aliye *et al.* 2015). In contrast to the numerous favorable changes obtained through mutagenesis, amino acid substitutions associated with loss of fluorescence remain incompletely characterized (Yang *et al.* 1996; Zacharias and Tsien 2006). Although deleterious or neutral mutations are not relevant for creating novel properties or improving GFP, they nevertheless might help in understanding GFP folding and chromophore maturation (Zacharias and Tsien 2006). With this in mind, we report here a collection of GFP loss-of-function mutations retrieved in a classical genetic screen for mutants displaying diminished fluorescence of a *GFP* reporter gene in *Arabidopsis thaliana*.

## Materials and Methods

### Plant line

In this study we used an *Arabidopsis thaliana* transgenic line in a Columbia (Col) ecotype background that is homozygous for a previously described target (*T*) locus encoding enhanced GFP protein (EGFP). This reporter gene is expressed primarily in shoot and root meristem regions and also in the hypocotyl (part of stem between seed leaves and root) of young seedlings (Kanno *et al.* 2008, 2010; Sasaki *et al.* 2012, 2015). EGFP was purchased from Clontech as plasmid pIRES2-EGFP. Relative to wild-type GFP, the EGFP protein contains a threonine at position 65 instead of serine and has an additional GTG (Val) codon after the initiating ATG (Met) codon to optimize translation initiation in eukaryotes. To conform to numbering of the wild-type GFP sequence (Zacharias and Tsien 2006), we consider the nucleotide and amino acids sequences of EGFP (referred to hereafter as simply GFP) without this additional Val residue throughout this article (nucleotide and amino acid sequences used are shown in Supporting Information, Figure S1). The GFP protein in the *T* line also has a 27 amino acid extension at the N-terminus because of the use of a cryptic promoter element upstream of the intended promoter in the transgene construct (Sasaki *et al.* 2015). These extra amino acids and mutations in this region are not considered in the present analysis, which focuses on loss-of-function mutations in the *GFP* protein coding sequence.

### EMS mutagenesis

Chemical mutagenesis with the alkylating agent ethane methylsulfonate (EMS) was performed as described previously (Kim *et al.* 2006). Approximately 40,000 EMS-treated seeds from the homozygous *T* line were sown on soil (M1 generation), grown to maturity, and allowed to self-fertilize to produce M2 seeds, which comprise the first generation in which recessive mutations can be homozygous and display a phenotype. The 1-wk-old to 2-wk-old M2 seedlings growing under sterile conditions on solid Murashige and Skoog (MS) medium in Petri dishes were screened for GFP fluorescence under a fluorescence stereo microscope. For seedlings showing very weak or no visible fluorescence, the *GFP* gene was sequenced to identify possible *gfp* loss-of-function mutations. From a screen of approximately 280,000 M2 seedlings (approximately seven M2 progeny were tested from each M1 plant) (Haughn and Somerville 1990; Jander *et al.* 2003), we retrieved 20 unique *gfp* mutants, which were combined for further analysis with eight additional unique, uncharacterized *gfp* mutants identified in a separate screen based on the same *GFP* reporter gene (Sasaki *et al.* 2012). Images of selected *gfp* mutants shown in Figure S2 were made using a Leica TCS LSI-III Confocal Microscope System.

### Sequencing the GFP gene

Genomic DNA was purified using a DNeasy Plant Mini Kit (QIAGEN) according to the manufacturer’s protocol. To amplify the *GFP* gene, PCR was performed using Ex Taq (Takara) and the following primers: GFP-R1, TATCTGGGAACTACTCACAC; and GFP-F1, GACAGAACTAATTATACCAG. The PCR conditions were as follows: 94° for 2 min followed by 35 cycles of 94° for 10 sec, 58° for 20 sec, and 72° for 90 sec, followed by a final extension for 7 min. The PCR fragments were purified using a GEL/PCR Purification Kit (Favorgen Corp., Taiwan) according to the manufacturer’s instruction followed by sequencing.

### Complementation tests

The original transgene construct encoding GFP that was used to obtain the *T* line (Kanno *et al.* 2008) was introduced either into binary vector pPZP221, which encodes resistance to gentamicin in plants (Hajdukiewicz *et al.* 1994), or into pCambia binary vector 1300 (http://www.cambia.org/daisy/cambia/585#dsy585_minimal_selection), in which the 35S promoter-hygromycin phosphotransferase gene was replaced by a phosphinothricin acetyltransferase gene under the control of the mannopine synthase promoter, which confers resistance to phosphinothricin in plants. These binary vectors were each used to transform the individual *gfp* mutants using the floral dip procedure (Clough and Bent 1998). Transformed seedlings were selected on solid MS medium containing either gentamicin or phosphinothricin and screened for *GFP* expression using a stereo fluorescence microscope. Complementation was considered successful when the level of visible GFP fluorescence exceeded that observed in the original *gfp* mutant. In particular, we observed successful complementation of mutations that were retrieved only once (T65I, G67S, R96C, G20D, G91S, C70Y, and V112M) (Table 1), confirming that the observed loss-of-fluorescence phenotypes were due to the respective amino acid substitutions in the GFP protein.

**Table 1:** List of *gfp* loss-of-function mutations identified in this screen

Amino Acid Change	Fluorescence*^a^*	Protein Accumulation*^b^*	Highly Conserved*^c^*	Reference*^e^*
**PTC*^d^***				
W 57* (G170A)				This study (batch 16); Sun *et al.* 2012
W 57* (G171A)	—	—		This study (batch 4)
Q 69*	—	—		Sasaki *et al.* 2012
Q 94*	—	Sterile		This study (batch 32)
Q 157*	—	—		Sasaki *et al.* 2012
Q 177*	—	—		Sasaki *et al.* 2012
Q 183*	—	—		Sasaki *et al.* 2012
Q 184*	—	Sterile		Sasaki *et al.* 2012
**Chromophore**				
T 65 I	Weak to moderate	Weak	No	This study (batch 35)
G 67 S	No	Weak	Yes	Sasaki *et al.* 2012
G 67 D	Weak	Weak	Yes	This study (batch 22), Sasaki *et al.* 2012; Sun *et al.* 2012
**Chromophore maturation**				
T 62 I	Negligible	No	No	Sasaki *et al.* 2012; Sun *et al.* 2012
R 96 C	No	WT	Yes	This study (batch 31)
R 96 H	No	WT	Yes	This study (batch 12); Sasaki *et al.* 2012
S 205 F	Weak	WT	No	Sasaki *et al.* 2012; Sun *et al.* 2012
E 222 K	Weak to moderate	Weak	Yes	This study (batches 17, 18)
**Protein stability—lid residues**				
G 20 D	No	No	Yes	This study (batch 38)
G 91 S	No	No	Yes	This study (batch 9)
G 91 D	No	No	Yes	This study (batch 3); Sasaki *et al.* 2012
G 127 D	Negligible	No	Yes	This study (batches 6, 17); Sun *et al.* 2012
**Protein stability—unknown function**				
G 31 D	Negligible	No	Yes	This study (batches 12, 31); Sasaki *et al.* 2012; Sun *et al.* 2012
G 33 D	Negligible	No	Yes	This study (batches 3, 28, 44)
G 35 S	Weak	Very weak	Yes	This study (batches 23, 27, 28)
G 40 D	Negligible	No	Yes	This study (batch 34); Sasaki *et al.* 2012
P 56 L	Negligible	No	No	This study (batch 25)
C 70 Y	Weak	Very weak	No	This study (batch 40)
A 110 V	Negligible	No	No	This study (batch 47); Sun *et al.* 2012
V 112 M	Weak	Weak	No	This study (batch 15)

Amino acid residues (column 1) are numbered according to wild-type GFP. In the present study and two previous studies (Sun *et al.* 2012 and Sasaki *et al.* 2012), a modified GFP containing an additional GTG (Val) codon in position 2 after the ATG (Met) start codon was used to optimize translation initiation in eukaryotes. However, for consistency of numbering with wild-type GFP, we have treated in this article the Val in position 2 as 1a (Zacharias and Tsien 2006).Therefore, amino acid numbers in the previous studies (Sun *et al.* 2012, Sasaki *et al.* 2012) are one higher compared to wild-type GFP. Two nucleotide changes (G170A and G171A) convert W57 into a PTC (Figure S1).

a In column 2, "no" indicates no visible fluorescence under fluorescence microscope; "negligible" indicates a very faint tinge of fluorescence in the hypocotyl; "weak" indicates barely visible fluorescence in hypocotyl; "weak to moderate" indicates visible fluorescence in the hypocotyl but not shoot meristem. The wild-type *T* line displays fluorescence in shoot and root meristem regions and in the hypocotyl in young seedlings (Figure S2).

b In column 3, the presence of a band on a Western blot probed with an antibody to GFP is indicated as "very weak," "weak," or wild-type (WT). "No" designates no visible band on the Western blot (Figure 2). "Sterile" indicates the mutant produced few or no seeds, presumably due to second site mutations unrelated to the mutation in *GFP* gene, and thus progeny seedlings were not available for Western blot analysis.

c Highly conserved residues are ones among the 23 identified as the most conserved in an analysis of sequences of 250 GFP-related proteins (Ong *et al.* 2011)

d Mutants containing premature stop codons (*) did not show any GFP fluorescence or protein band on the Western blot (indicated by dashes in columns 2 and 3).

e Batch number refers to one of 54 batches of pooled M2 seeds collected from the approximately 40,000 M1 plants grown from mutagenized seed.

### Western blot procedure to detect GFP protein

Approximately 100 mg of 2-wk-old seedlings of the M2 or M3 generation (grown under sterile conditions on solid MS medium) were ground in liquid nitrogen in a 1.5-ml Eppendorf tube with pellet pestles (Sigma, Z359947) to a fine powder. The powder was then re-suspended in 150 µl of PEB buffer (50 mM Tris, 400 mM KCl, 2.5 mM MgCl_2_, 1 mM EDTA, 1 mM DDT, 0.1% Triton-X, 1X protease Inhibitor, Roche 5056489001), vortexed three times for 20 sec each (between intervals place tube back on ice), and centrifuged at 15,000 rpm at 4° for 10 min. Then, 100 µl of supernatant was combined with 100 µl PEB buffer (without KCl) in a new 1.5-ml tube. The amount of protein was quantified using the Bradford protein assay and the solution was then stored at −20°. From each sample, 20 µg of protein were separated by sodium dodecylsulfate (SDS) polyacrylamide gel electrophoresis (PAGE) using 12% acrylamide gels. After SDS-PAGE, proteins were transferred onto a polyvinylidene difluoride (PVDF) membrane (Bio-Rad). The membrane was then incubated in a plastic container with blocking reagent [10% w/v skim milk in 0.02% Tween 20-TBS (TBST)] for 1 hr at room temperature with gentle rocking. GFP protein was detected by incubating the membrane with the 1:1000 dilution of anti-GFP antibody (Roche) at 4° overnight. The membrane was then washed three times using 0.02% TBST for 5 min, after which the secondary antibody was added [1:5000 anti-mouse IgG-HRP (Bio-Rad)] and incubated with the membrane for 1 hr at room temperature. The signal was detected using a BioSpectrum Imaging System (UVP, Jena) after washing two times for 5 min with TBST and applying enhanced chemiluminescence (ECL) substrates (Advansta K-12045D50). The membrane was then stripped clean with Western Blot Stripping Buffer (Thermo PIE21059) and re-probed with the 1:1000 diluted monoclonal anti-α-tubulin (Sigma, T6199) at 4° overnight. The following day, the membrane was washed with 0.02% TBST, the secondary antibody was added [1:5000 anti-mouse IgG-HRP (Bio-Rad)], and the membrane was incubated with gentle agitation for 1 hr at room temperature. The signal was detected using a BioSpectrum Imaging System (UVP, Jena) as described above.

### Detection of GFP mRNA by RT-PCR

Total RNA was extracted from approximately 2-wk-old seedlings (grown under sterile conditions on solid MS medium) using a Plant Total RNA Miniprep Purification Kit (GeneMark) followed by RQ1 DNase (Promega) treatment, according to the manufacturer’s instructions. cDNA was synthesized using Transcriptor First Strand cDNA Synthesis Kit (Roche) using an oligonucleotide d(T) primer and 1 μg of total RNA. To detect the *GFP* transcripts, 1 μl of cDNA was used for RT-PCR with the following primers: forward primer, 5′-CACCTACGGCAAGCTGACCCTG-3′ and reverse primer, 5′- TTTACTTGTACAGCTCGTCC-3′. The PCR conditions were as follows: 94° for 2 min followed by 27 cycles of 94° for 10 sec, 58° for 20 sec, and 72° for 1 min, and finally 72° for 7 min.

### Data availability

Seeds of mutants listed in Table 1 are available upon request.

## Results and Discussion

A transgenic *Arabidopsis* line (*T* line) that is homozygous for a stably expressed enhanced *GFP* (EGFP) reporter gene (referred to hereafter as simply GFP) (Kanno *et al.* 2008, 2010) was used in this screen. Seeds of the *T* line were treated with EMS and M2 seedlings, which represent the first generation when a recessive mutation can be homozygous, were visualized under a fluorescence stereo microscope to screen for GFP-negative or very weak phenotypes. DNA was isolated from these plants and the *GFP* gene was sequenced to identify possible loss-of-function mutations. From this screen we retrieved 20 unique *gfp* mutants, which were combined for further analysis with eight additional unique, uncharacterized *gfp* mutants recovered in a separate screen based on the same *GFP* reporter gene (Sasaki *et al.* 2012) (Table 1).

EMS induces almost exclusively C-to-T changes resulting in C/G to T/A transition mutations (Kim *et al.* 2006), thus limiting our investigation to this type of mutation. A previous analysis of the structure of more than 250 GFP-like proteins in the Protein Databank identified 23 highly conserved amino acids (Ong *et al.* 2011), 21 of which are present in the GFP protein used in this study (Figure S1). Many of the most highly conserved residues are situated at the lids on the top and bottom of the β-barrel and at bends between the β-sheets (Ong *et al.* 2011; Zimmer *et al.* 2014). The purpose of the lids is unclear, but the low variability displayed by these regions in GFP-like proteins suggests an important role in folding, stability, or other aspect of GFP function (Ong *et al.* 2011; Stepanenko *et al.* 2013; Zimmer *et al.* 2014). Our screen retrieved mutations resulting in substitutions of 11 of the 21 highly conserved amino acids present in the GFP protein used in this study (Table 1). As described below, the codons of some highly conserved amino acids in the GFP protein are not susceptible to mutagenesis by EMS. The mutations we identified can be placed into five categories and considered in the context of the 23 most highly conserved amino acids in GFP-like proteins (Ong *et al.* 2011).

### Premature termination codons

Removing only seven amino acids from the C terminus and one from the N terminus destroys GFP function (Yang *et al.* 1996). In the *GFP* coding sequence (Figure S1), premature termination codons (PTC) can be induced by EMS only at codons for glutamine and tryptophan (Table S1). The fact that we retrieved mutations affecting the single tryptophan in the GFP protein (Trp57) and six of eight glutamine residues (only Gln80 and Gln204 were not mutated in our screens; Figure S1) suggests that the combined screens approached saturation. The truncations resulting from these PTCs far exceed those tolerated at the N and C termini of GFP, which is consistent with the lack of GFP protein accumulation (Figure 2A) and visible fluorescence in PTC mutant seedlings (Table 1).

### Chromophore

**Figure 1:**
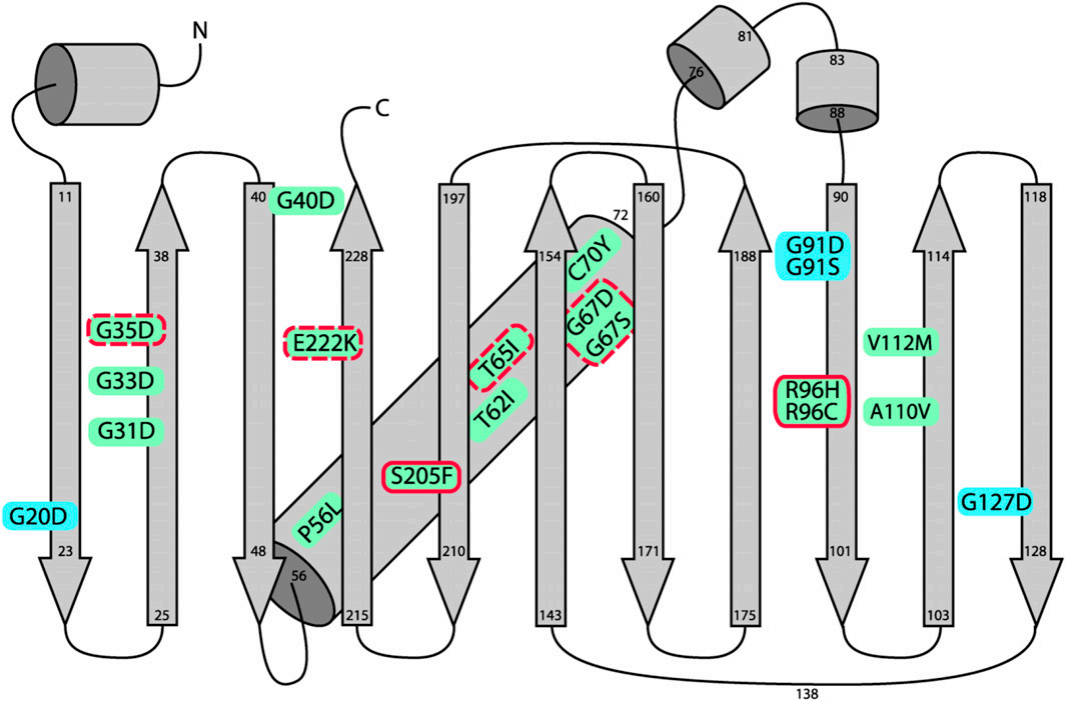
Overall GFP fold and positions of amino acid substitutions leading to loss of fluorescence. A schematic depiction of the overall fold of Thr65-GFP protein (Yang *et al.* 1996) is redrawn here. The vertical arrows indicate the 11 β strands of the β-barrel structure. Amino acid residue numbers at the base and tips of the arrows indicate the beginning and ends of secondary structural elements. The chromogenic tripeptide (Thr65-Tyr66-Gly67) is positioned on an internal α-helix (diagonal cylinder) extending from amino acids 56 to 72. Amino acid substitutions identified in our screen that lead to losses of fluorescence are indicated. Solid red outlines denote substitutions causing defects in chromophore formation without substantial reductions in GFP protein accumulation. Dotted red outlines indicate substitutions resulting in lowered levels of GFP protein accumulation relative to wild-type. For the remaining substitutions, no GFP protein was detected by Western blotting under the conditions used. Lid residues at the N and C termini (G91 and G127) and the opposite side (G20), which is referred to as the "top" of the barrel (Zimmer *et al.* 2014), are highlighted in blue.

**Figure 2:**
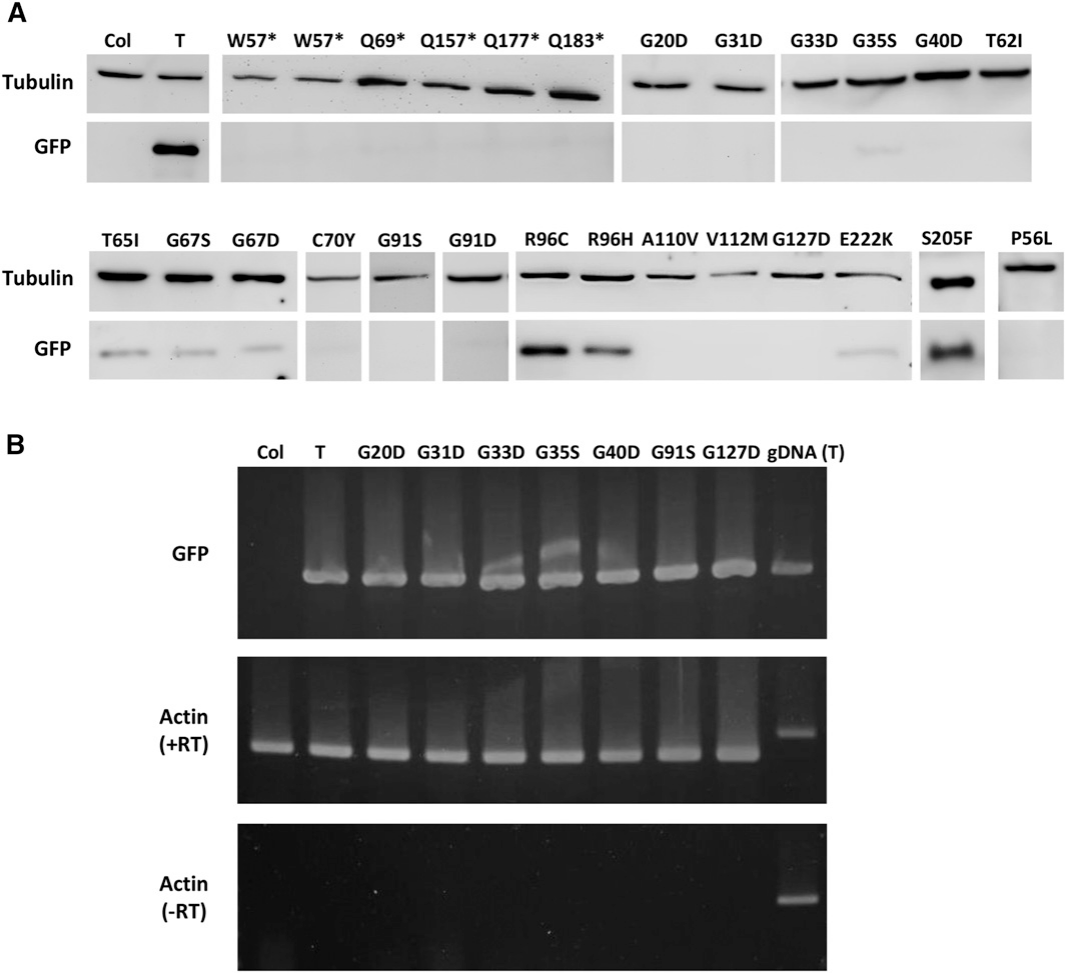
Analysis of GFP expression in gfp loss-of-function mutants. (A) GFP protein in gfp mutants identified in our screen was analyzed by Western blotting (bottom) using tubulin as a loading control (top). The amino acid substitution or PTC (asterisks) in a given gfp loss-of-function mutant is indicated at the top of each lane. The original nonmutagenized target line (T) and wild-type Arabidopsis (Col) were used routinely as positive and negative controls, respectively, for all blots but representative results are shown here only once. Findings from only the fertile PTC mutants (Table 1) are shown. The two W57* samples indicate independent mutations (G170A and G171A) in the GFP coding sequence that change a tryptophan codon into a PTC (Figure S1). (B) Semi-quantitative RT-PCR was used to detect GFP RNA in gfp mutants resulting from substitutions in the indicated highly conserved glycine residues. These mutants did not accumulate significant amounts of GFP protein (Figure 2) but contained comparable levels of GFP RNA as the nonmutagenized T line (upper panel). Actin was used as an internal control (middle panel). No reverse-transcriptase (-RT) is shown as technical control. "gDNA" indicates genomic DNA isolated from the nonmutagenized T line.

From the chromogenic tripeptide (Thr65-Tyr66-Gly67), we recovered substitutions of Thr65 (T65I) and Gly67 (G67S and G67D) (Table 1, Figure 1). Because the only EMS-induced mutation of tyrosine codons produces a silent mutation (Table S1), it was not possible to obtain substitutions of highly conserved Tyr66 in our screen. Gly67 is also among the 23 highly conserved amino acids in GFP-related proteins (Ong *et al.* 2011). Gly67 mutant plants did not show visible GFP fluorescence (Table 1, Figure S2), although they did accumulate detectable, albeit reduced, levels of GFP protein (Figure 2A), suggesting that the G67 substitutions compromise to some extent GFP protein stability. The lack of visible fluorescence despite detectable protein accumulation is consistent with previous results showing that substituting Gly67 with any other amino acid prevents chromophore formation (Lemay *et al.* 2008; Stepanenko *et al.* 2013). Glycine is unique in having H as a side chain, in contrast to other amino acids that have a carbon, endowing glycine with exceptional conformational flexibility (Betts and Russell, 2003). Thus, glycine is the only amino acid at position 67 that permits formation of a kinked internal α-helix, which places Gly67 close to the residue at position 65 for nucleophilic attack during chromophore synthesis (Lemay *et al.* 2008; Stepanenko *et al.* 2013).

Position 65 is considered the most variable of the chromogenic tripeptide (Stepanenko *et al.* 2013). Accordingly, the T65I substitution we identified reduced but did not eliminate visible fluorescence in mutant seedlings (Figure S2). Similar to the G67 substitutions, the amount of GFP protein in the T65I mutant was lowered but not abolished (Figure 2A), implying reduced GFP protein stability in addition to probable inhibition of chromophore formation in this mutant.

### Chromophore maturation

Chromophore synthesis requires a network of polar interactions involving amino acids of the chromogenic tripeptide as well as several closely apposed amino acids, including four that we identified in our screen: Glu222 and Arg96, which are among the 23 most highly conserved amino acids in GFP-related proteins (Ong *et al.* 2011), as well as Thr62 and Ser205 (Yang *et al.* 1996; Ormö *et al.* 1996). In particular, Arg96, which acts as an electrostatic catalyst, and Glu222, which behaves as a base catalyst, are considered critical features for chromophore synthesis (Stepanenko *et al.* 2013).

The E222K substitution we identified did not eliminate fluorescence but resulted in weak to moderate GFP fluorescence in seedlings (Table 1, Figure S2). In accordance with this finding, an E222G mutant has been reported previously to fluorescence, indicating that Glu222 is not absolutely required for chromophore formation (Lemay *et al.* 2008). Only low levels of GFP protein were observed in the E222K mutant (Figure 2A), suggesting an impact of the E222K substitution on GFP protein stability. In addition to a catalytic role, Glu222 has been reported to have a stabilizing function by contributing to the rigidity of the chromophore cavity (Royant and Noirclerc-Savoye 2011; Stepanenko *et al.* 2013).

The S205F mutant displayed weak to moderate GFP fluorescence in seedlings (Table S1) despite the accumulation of nearly wild-type levels of GFP protein (Figure 2A). These findings are consistent with the role of Ser205 in the hydrogen bonding network required for chromophore maturation (Yang *et al.* 1996).

The most extreme losses of fluorescence (Figure S2) accompanied by nearly wild-type levels of GFP protein (Figure 2A) were observed with the Arg96 substitutions: R96C and R96H (Table 1). Although a R96C substitution has been reported previously to fluoresce (Lemay *et al.* 2008), our findings with this substitution as well as R96H suggest that Arg96 is essential for chromophore formation and hence GFP fluorescence in plants. Arg96 forms a hydrogen bond with T62 (Ormö *et al.* 1996) and, in our study, a T62I substitution resulted in decreased fluorescence in mutant seedlings (Table 1). However, the diminished fluorescence was likely a reflection of decreased protein stability because the T62I mutant did not accumulate appreciable amounts of GFP protein (Figure 2A).

### Highly conserved glycine residues and GFP protein stability

There are 22 glycine residues in the GFP protein (Figure S1). Along with substitutions of Gly67 in the chromogenic tripeptide, loss-of-function substitutions were recovered for seven additional glycines, all of which are among the 23 most highly conserved amino acids in GFP-related proteins (Ong *et al.* 2011) (Table 1). The roles of these glycine residues have so far been unclear (Stepanenko *et al.* 2013), but our data suggest an essential role in GFP protein stability.

We recovered substitutions in three highly conserved "lid" residues in our screen: Gly91 is situated at the lid of the N and C termini, which are in close proximity at the same end of the barrel (Figure 1), whereas Gly20 and Gly127 are located on the opposite lid, which is referred to as the "top" of the barrel (Ong *et al.* 2011; Zimmer *et al.* 2014). Consistent with the fact that glycines can reside in parts of proteins that are unable to accommodate other amino acids, such as tight turns in structures (Betts and Russell, 2003), these three lid residues as well as Gly40, also identified in our screen, are all present in the vicinity of bends representing transitions from β-strands to loops in the folding pattern of GFP (Figure 1) (Zimmer *et al.* 2014).

Additional essential glycines identified in the screen include Gly31, Gly33, and Gly35, which are located on the second β-strand of the 11 β-strand barrel (Figure 1). These three glycines are the only conserved residues residing on β-strands that are not involved in chromophore formation and their function is uncertain (Ong *et al.* 2011; Stepanenko *et al.* 2013). With the exception of G35S, which showed very weak fluorescence in seedlings (Figure S2) and very low levels of GFP protein (Figure 2A), all of the glycine substitutions resulted in a lack of both visible fluorescence (Table 1) and detectable GFP protein (Figure 2A) despite normal transcription of *GFP* mRNA (Figure 2B). Our results implicate these highly conserved glycine residues in facilitating GFP protein folding and stability, which is consistent with a previous computational analysis of the structures of GFP and related proteins (Zimmer *et al.* 2014).

### Nonconserved residues contributing to GFP protein folding or stability

Four loss-of-function mutations in residues that are not among the most highly conserved amino acids of GFP-related proteins were identified in our screen (Table 1). P56L, A110V, and V112M substitutions are likely to represent residues important for folding or structural stability because GFP protein did not accumulate to detectable levels in the respective mutants (Figure 2A). Pro56 is located at the beginning of the internal α-helix containing the chromogenic tripeptide (Figure 1) and, together with several other proline residues, is thought to be important for maintaining kinks in the α-helical backbone, which are necessary for chromophore synthesis (Stepanenko *et al.* 2013). Ala110 and Val112 are not at transitions in the secondary structure (Figure 1), and they are not among the 23 most highly conserved amino acid residues (Table 1) (Ong *et al.* 2011). The details of their contributions to GFP protein folding and stability thus remain to be clarified.

A C70Y substitution also significantly decreased accumulation of GFP protein (Figure 2A), resulting in substantially reduced visible fluorescence in seedlings (Figure S2). Cys70 is relatively close to the chromophore and to a transition between the internal α-helix and a loop (Figure 1). In contrast to the C70Y loss-of-function substitution, a C70V substitution was found to improve folding properties of a GFP variant (Zapata-Hommer and Griesbeck 2003). The different effects of the two mutations suggest that different substitutions of Cys70 can have either positive or negative effects on GFP folding and stability.

### Highly conserved residues not identified in this genetic screen

We recovered loss-of-function substitutions in 11 of the 21 highly conserved residues found in the GFP protein used in this study (Ong *et al.* 2011) (Table 1, Figure S1). The remaining 10 highly conserved residues include Tyr66 of the chromogenic tripeptide, Phe27, Phe130, Leu53, and Ile136 (Figure S1). As described above, EMS-induced mutation of tyrosine codons produce only a silent change, and the same holds for the codons of phenylalanine, leucine, and isoleucine (Table S1). Therefore, EMS-induced mutagenesis cannot be used to probe the contributions of these highly conserved amino acids to GFP fluorescence and stability.

Assuming that conservation implies an essential function, it is not clear why we did not recover loss-of-function mutations in the final five highly conserved residues: Val55, Asp102, Gly104, Gly134, and Pro196 (Figure S1) (Ong *et al.* 2011). The codons of these amino acids are in principle targets of EMS-induced point mutations that result in amino acid substitutions (Table S1). In particular, the failure to recover substitutions of G104 and G134 is unusual, because mutations altering seven other highly conserved glycines were retrieved in the screen, in most cases more than once (Table 1). These results may indicate that our screen is not yet saturated or that substitutions in these highly conserved residues do not lead to losses of GFP fluorescence that are detectable by our visual screening procedure.

### Summary

We identified a collection of GFP loss-of-function mutations that provide information about amino acids important for chromophore maturation and GFP protein stability. The mutations we identified result in substitutions of 11 of the 21 most conserved amino acids in the GFP protein used in this study as well as in seven less conserved amino acids. Mutations leading to substitutions of highly conserved Arg96 required for chromophore formation appear to substantially decrease or eliminate GFP fluorescence by impairing chromophore formation without dramatically affecting protein stability. By contrast, other mutations result in amino acid substitutions that apparently compromise protein stability and accumulation, hence leading to visible reductions in fluorescence. These substitutions affect the absolutely conserved Gly67 of the chromogenic tripeptide, seven highly conserved glycines in the lids and secondary structural transitions of the β-barrel structure (Gly20, Gly40, Gly91, Gly127) and in the second β-strand (Gly31, Gly33, Gly35), and four nonconserved residues not previously implicated in GFP protein stability (Pro56, Cys70, Ala110, Val112). We also identified substitutions of amino acids involved in chromophore maturation (Thr62 and highly conserved Glu222) that are likely to influence GFP protein stability in addition to impairing fluorescence, presumably because of unsuccessful chromophore formation. These genetic findings support results from biochemical and structural analyses and contribute to a fuller understanding of amino acids, particularly numerous highly conserved glycine residues with previously unknown roles, that are essential for the function and stability of the GFP protein in a higher eukaryotic organism.

## Supplementary Material

Supporting Information
