# Supplementary material for: GFP Loss-of-Function Mutations in Arabidopsis thaliana
Source: G3 (Bethesda). 2015 Jul 6;5(9):1849–55. doi: 10.1534/g3.115.019604 (PMC4555221; doi:10.1534/g3.115.019604)
Supplement: Supporting Information [file supp_5_9_1849__index.html]

GFP Loss-of-Function Mutations in Arabidopsis thaliana — Supporting Information 

# GFP Loss-of-Function Mutations in *Arabidopsis thaliana*

## Supporting Information for Fu *et al.*, 2015

**Files in this Data Supplement:**

- Supporting Information - Figures S1-S2 and Table S1 (PDF, 1 MB)
- Figure S1 - *GFP* DNA and amino acid sequences showing mutations retrieved in this study (yellow highlights) and resulting amino acid changes (black arrowheads). (PDF, 349 KB)
- Figure S2 - Photos of selected *gfp* mutant seedlings (focusing on the hypocotyl region approximately two weeks after germination on solid, sterile MS medium) showing complete or partial losses of fluorescence (top panels) compared to the non-mutagenized *T* line containing the GFP reporter gene (upper right). (PDF, 351 KB)
- Table S1 - Amino acid codons relevant to this study. (PDF, 286 KB)
