# Supplementary material for: GFP Loss-of-Function Mutations in Arabidopsis thaliana
Source: G3 (Bethesda). 2015 Jul 6;5(9):1849–55. doi: 10.1534/g3.115.019604 (PMC4555221; doi:10.1534/g3.115.019604)
Supplement: Supporting Information [file supp_g3.115.019604_019604SI.pdf]

G3: Genes | Genomes | Genetics

GFP loss-of-function mutations in *Arabidopsis thaliana*

Jason L. Fu, Tatsuo Kanno, Shih-Chieh Liang, Antonius J.M. Matzke and Marjori Matzke

Institute of Plant and Microbial Biology, Academia Sinica

128, Section 2, Academia Road, Nangang District

Taipei 115, Taiwan

Corresponding author:

Antonius J.M. Matzke

Institute of Plant and Microbial Biology, Academia Sinica

128, Sec. 2 Academia Rd., Nangang

Taipei 115, Taiwan

Tel: +886-2-2787-1135, Email: antoniusmatzke@gate.sinica.edu.tw

Co-corresponding author:

Marjori Matzke

Institute of Plant and Microbial Biology, Academia Sinica

128, Sec. 2 Academia Rd., Nangang

Taipei 115, Taiwan

Tel: +886-2-2787-1135, Email: marjorimatzke@gate.sinica.edu.tw

DOI: 10.1534/g3.115.019604

Figure S1, Fu et al.

```
DNA:   atgagcaagggcgaggagctgttcaccggggtggtgcccatcctggtcgagctggacggc  60
A.A.:  ·M··S··K··G··E··E··L··F··T··G··V··V··P··I··L··V··E··L··D··G·  20
                                                              D

DNA:   gacgtaaacggccacaagttcagcgtgtccggcgagggcgagggcgatgccacctacggc  120
A.A.:  ·D··V··N··G··H··K··F··S··V··S··G··E··G··E··G··D··A··T··Y··G·  40
                                      D     D     S              D

DNA:   aagctgaccctgaagttcatctgcaccaccggcaagctgcccgtgccctggcccaccctc  180
A.A.:  ·K··L··T··L··K··F··I··C··T··T··G··K··L··P··V··P··W··P··T··L·  60
                                                    L  *  *

DNA:   gtgaccaccctgacctacggcgtgcagtgcttcagccgctaccccgaccacatgaagcag  240
A.A.:  ·V··T··T··L··T··Y··G··V··Q··C··F··S··R··Y··P··D··H··M··K··Q·  80
          I        I     S  D  *  Y

DNA:   cacgacttcttcaagtccgccatgcccgaaggctacgtccaggagcgcaccatcttcttc  300
A.A.:  ·H··D··F··F··K··S··A··M··P··E··G··Y··V··Q··E··R··T··I··F··F·  100
                                   S  D        *     C  H

DNA:   aaggacgacggcaactacaagacccgcgccgaggtgaagttcgagggcgacaccctggtg  360
A.A.:  ·K··D··D··G··N··Y··K··T··R··A··E··V··K··F··E··G··D··T··L··V·  120
                                  V     M

DNA:   aaccgcatcgagctgaagggcatcgacttcaaggaggacggcaacatcctggggcacaag  420
A.A.:  ·N··R··I··E··L··K··G··I··D··F··K··E··D··G··N··I··L··G··H··K·  140
                         D

DNA:   ctggagtacaactacaacagccacaacgtctatatcatggccgacaagcagaagaacggc  480
A.A.:  ·L··E··Y··N··Y··N··S··H··N··V··Y··I··M··A··D··K··Q··K··N··G·  160
                                                     *

DNA:   atcaaggtgaacttcaagatccgccacaacatcgaggacggcagcgtgcagctcgccgac  540
A.A.:  ·I··K··V··N··F··K··I··R··H··N··I··E··D··G··S··V··Q··L··A··D·  180
                                                     *

DNA:   cactaccagcagaacacccccatcggcgacggccccgtgctgctgcccgacaaccactac  600
A.A.:  ·H··Y··Q··Q··N··T··P··I··G··D··G··P··V··L··L··P··D··N··H··Y·  200
             *  *

DNA:   ctgagcacccagtccgccctgagcaaagaccccaacgagaagcgcgatcacatggtcctg  660
A.A.:  ·L··S··T··Q··S··A··L··S··K··D··P··N··E··K··R··D··H··M··V··L·  220
                   F

DNA:   ctggagttcgtgaccgccgccgggatcactctcggcatggacgagctgtacaagtaa  717
A.A.:  ·L··E··F··V··T··A··A··G··I··T··L··G··M··D··E··L··Y··K··*·  238
          K
```

Figure S1: *GFP* DNA and amino acid sequences showing mutations retrieved in this study (yellow highlights) and resulting amino acid changes (black arrowheads). The 23 most highly conserved amino acids in GFP-like proteins are indicated in red letters (Ong *et al*. 2011). Two glutamine residues that were not mutated in our screens (Q80 and Q204) are shown in blue.

Ong, W.J., S. Alvarez, I.E. Leroux, R.S. Shahid, A.A. Samma, P. Peshkepija, A.L. Morgan, S. Mulcahy and M. Zimmer, 2011 Function and structure of GFP-like proteins in the protein data bank. Mol. Biosyst. 7: 984-992.

Figure S2, Fu et al.

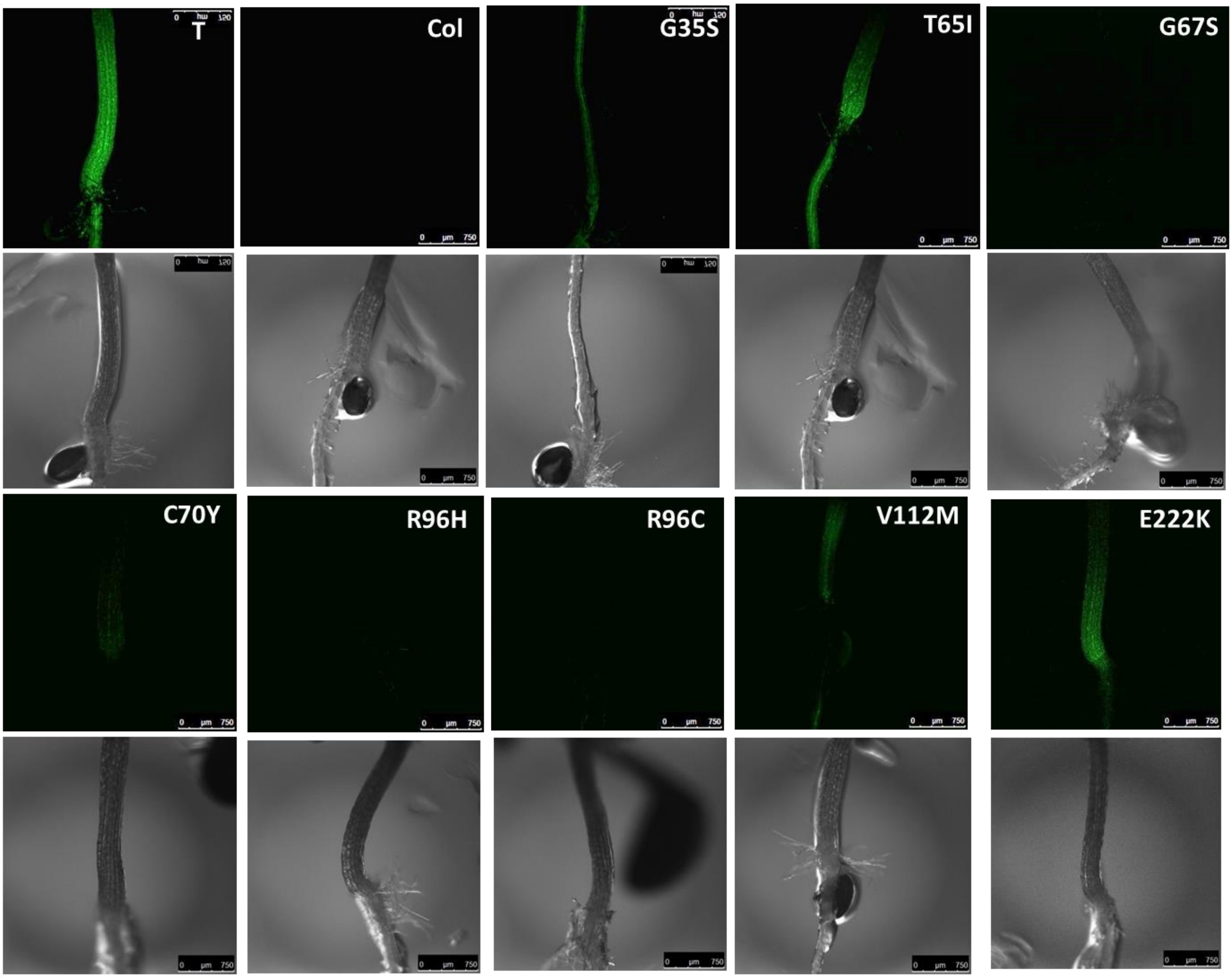

Figure S2: Photos of selected *gfp* mutant seedlings (focusing on the hypocotyl region approximately two weeks after germination on solid, sterile MS medium) showing complete or partial losses of fluorescence (top panels) compared to the non-mutagenized *T* line containing the GFP reporter gene (upper right). Images of the same seedlings taken in white light are shown below each fluorescence image. Col is wild-type *Arabidopsis thaliana* ecotype Columbia.

Table S1, Fu et al.

Table S1: Amino acid codons relevant to this study. EMS induces C/G to A/T mutations. PTC, premature termination codon.

**Amino acid**	**Codon usage in *GFP* reporter gene**	**Changes possible by EMS treatment**	**Resulting amino acid substitution**	
Tryptophan (Trp, W)	TGG	TGA	Stop (PTC)	
		TAG	Stop (PTC)	
Glutamine (Gln, Q)	CAA	TAA	Stop (PTC)	
	CAG	TAG	Stop(PTC)	
Tyrosine (Tyr, Y)	TAC	TAT	silent	
Phenylalanine (Phe, F)	TTC	TTT	silent	
Leucine (Leu, L)	CTG	TTG	silent	
		CTA	silent	
Isoleucine (Ile, I)	ATC	ATT	silent	
Valine (Val, V)	GTG	ATG	Methionine (Met, M)	
		GTA	silent	
Glutamic Acid (Glu, D)	GAC	AAC	Asparagine (Asn, N)	
		GAT	Aspartic acid (Asp, D)	
Proline (Pro, P)	CCC	TCC	Serine (Ser, S)	
		CTC	Leucine (Leu, L)	
		CCT	silent	
Glycine (Gly, G)	GGC	AGC	Serine (Ser, S)	
		GAC	Aspartic acid (Asp, D)	
		GGT	silent	
