# Supplementary material for: GFP Loss-of-Function Mutations in Arabidopsis thaliana
Source: G3 (Bethesda). 2015 Jul 6;5(9):1849–55. doi: 10.1534/g3.115.019604 (PMC4555221; doi:10.1534/g3.115.019604)
Supplement: Supporting Information [file supp_g3.115.019604_FigureS1.pdf]

Figure S1, Fu et al.

```
DNA:    atgagcaagggcgaggagctgttcaccggggtggtgcccatcctggtcgagctggacggc  60
A.A.:   ·M··S··K··G··E··E··L··F··T··G··V··V··P··I··L··V··E··L··D··G·  20
                                                                 D

DNA:    gacgtaaacggccacaagttcagcgtgtccggcgagggcgagggcgatgccacctacggc  120
A.A.:   ·D··V··N··G··H··K··F··S··V··S··G··E··G··E··G··D··A··T··Y··G·  40
                                       D     D     S              D

DNA:    aagctgaccctgaagttcatctgcaccaccggcaagctgcccgtgccctggcccaccctc  180
A.A.:   ·K··L··T··L··K··F··I··C··T··T··G··K··L··P··V··P··W··P··T··L·  60
                                                      L  *  *

DNA:    gtgaccaccctgacctacggcgtgcagtgcttcagccgctaccccgaccacatgaagcag  240
A.A.:   ·V··T··T··L··T··Y··G··V··Q··C··F··S··R··Y··P··D··H··M··K··Q·  80
            I        I    S  D    *  Y

DNA:    cacgacttcttcaagtccgccatgcccgaaggctacgtccaggagcgcaccatcttcttc  300
A.A.:   ·H··D··F··F··K··S··A··M··P··E··G··Y··V··Q··E··R··T··I··F··F·  100
                                      S  D       *    C  H

DNA:    aaggacgacggcaactacaagacccgcgccgaggtgaagttcgagggcgacaccctggtg  360
A.A.:   ·K··D··D··G··N··Y··K··T··R··A··E··V··K··F··E··G··D··T··L··V·  120
                                       V     M

DNA:    aaccgcatcgagctgaagggcatcgacttcaaggaggacggcaacatcctggggcacaag  420
A.A.:   ·N··R··I··E··L··K··G··I··D··F··K··E··D··G··N··I··L··G··H··K·  140
                           D

DNA:    ctggagtacaactacaacagccacaacgtctatatcatggccgacaagcagaagaacggc  480
A.A.:   ·L··E··Y··N··Y··N··S··H··N··V··Y··I··M··A··D··K··Q··K··N··G·  160
                                                        *

DNA:    atcaaggtgaacttcaagatccgccacaacatcgaggacggcagcgtgcagctcgccgac  540
A.A.:   ·I··K··V··N··F··K··I··R··H··N··I··E··D··G··S··V··Q··L··A··D·  180
                                                        *

DNA:    cactaccagcagaacacccccatcggcgacggccccgtgctgctgcccgacaaccactac  600
A.A.:   ·H··Y··Q··Q··N··T··P··I··G··D··G··P··V··L··L··P··D··N··H··Y·  200
               *  *

DNA:    ctgagcacccagtccgccctgagcaaagaccccaacgagaagcgcgatcacatggtcctg  660
A.A.:   ·L··S··T··Q··S··A··L··S··K··D··P··N··E··K··R··D··H··M··V··L·  220
                     F

DNA:    ctggagttcgtgaccgccgccgggatcactctcggcatggacgagctgtacaagtaa  717
A.A.:   ·L··E··F··V··T··A··A··G··I··T··L··G··M··D··E··L··Y··K··*·  238
            K
```

Figure S1*: GFP* DNA and amino acid sequences showing mutations retrieved in this study (yellow highlights) and resulting amino acid changes (black arrowheads). The 23 most highly conserved amino acids in GFP-like proteins are indicated in red letters (Ong *et al*. 2011). Two glutamine residues that were not mutated in our screens (Q80 and Q204) are shown in blue.

Ong, W.J., S. Alvarez, I.E. Leroux, R.S. Shahid, A.A. Samma, P. Peshkepija, A.L. Morgan, S. Mulcahy and M. Zimmer, 2011 Function and structure of GFP-like proteins in the protein data bank. Mol. Biosyst. 7: 984-992.
