# Supplementary material for: GFP Loss-of-Function Mutations in Arabidopsis thaliana
Source: G3 (Bethesda). 2015 Jul 6;5(9):1849–55. doi: 10.1534/g3.115.019604 (PMC4555221; doi:10.1534/g3.115.019604)
Supplement: Supporting Information [file supp_g3.115.019604_FigureS2.pdf]

Figure S2, Fu et al.

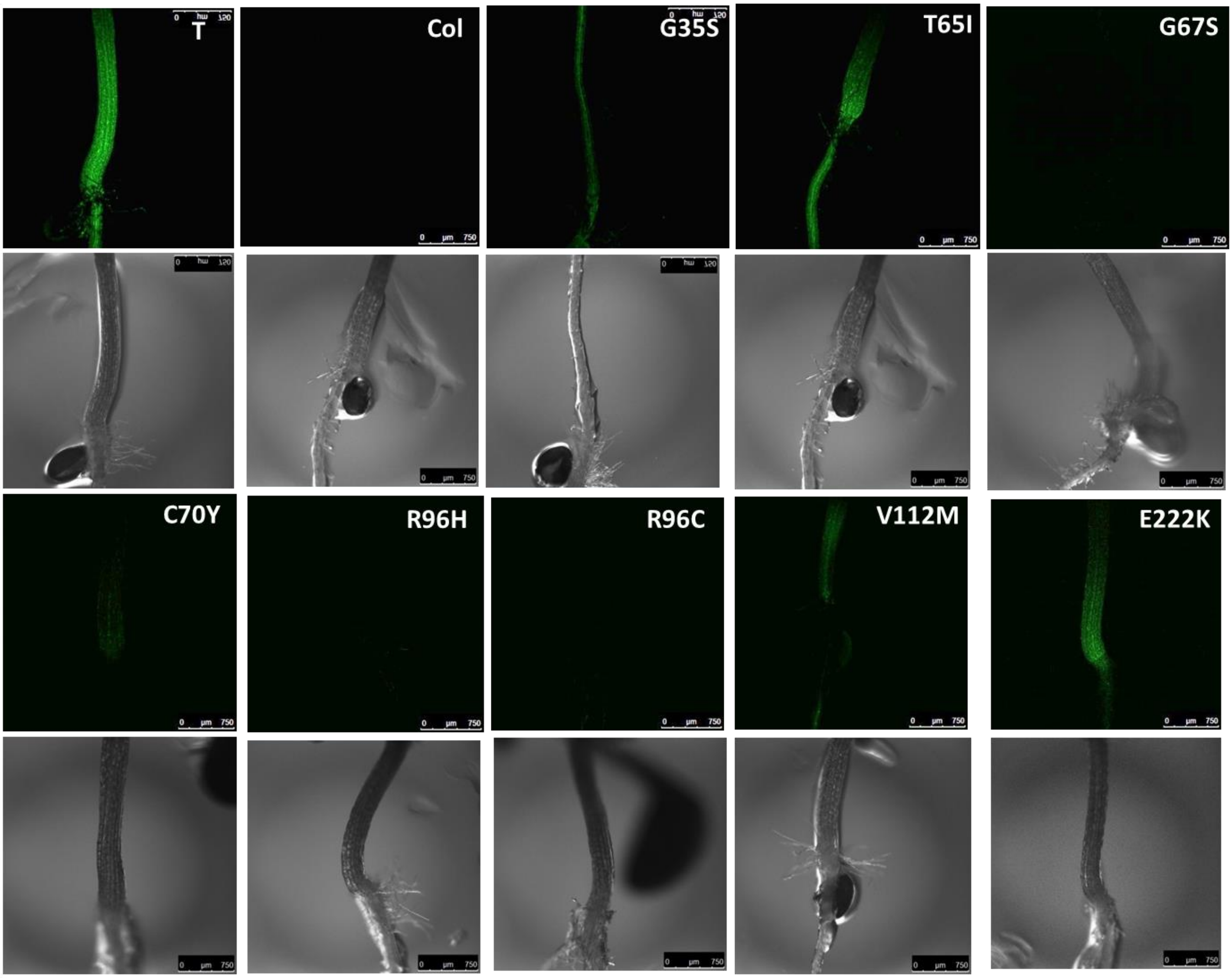

Figure S2: Photos of selected *gfp* mutant seedlings (focusing on the hypocotyl region approximately two weeks after germination on solid, sterile MS medium) showing complete or partial losses of fluorescence (top panels) compared to the non-mutagenized *T* line containing the GFP reporter gene (upper right). Images of the same seedlings taken in white light are shown below each fluorescence image. Col is wild-type *Arabidopsis thaliana* ecotype Columbia.
