# Supplementary material for: GFP Loss-of-Function Mutations in Arabidopsis thaliana
Source: G3 (Bethesda). 2015 Jul 6;5(9):1849–55. doi: 10.1534/g3.115.019604 (PMC4555221; doi:10.1534/g3.115.019604)
Supplement: Supporting Information [file supp_g3.115.019604_TableS1.pdf]

Table S1, Fu et al.

Table S1: Amino acid codons relevant to this study. EMS induces C/G to A/T mutations. PTC, premature termination codon.

**Amino acid**	**Codon usage in *GFP* reporter gene**	**Changes possible by EMS treatment**	**Resulting amino acid substitution**	
Tryptophan (Trp, W)	TGG	TGA	Stop (PTC)	
		TAG	Stop (PTC)	
Glutamine (Gln, Q)	CAA	TAA	Stop (PTC)	
	CAG	TAG	Stop(PTC)	
Tyrosine (Tyr, Y)	TAC	TAT	silent	
Phenylalanine (Phe, F)	TTC	TTT	silent	
Leucine (Leu, L)	CTG	TTG	silent	
		CTA	silent	
Isoleucine (Ile, I)	ATC	ATT	silent	
Valine (Val, V)	GTG	ATG	Methionine (Met, M)	
		GTA	silent	
Glutamic Acid (Glu, D)	GAC	AAC	Asparagine (Asn, N)	
		GAT	Aspartic acid (Asp, D)	
Proline (Pro, P)	CCC	TCC	Serine (Ser, S)	
		CTC	Leucine (Leu, L)	
		CCT	silent	
Glycine (Gly, G)	GGC	AGC	Serine (Ser, S)	
		GAC	Aspartic acid (Asp, D)	
		GGT	silent	
